# Identification of the Immune-Related Genes in Tumor Microenvironment That Associated With the Recurrence of Head and Neck Squamous Cell Carcinoma

**DOI:** 10.3389/fcell.2021.723721

**Published:** 2021-08-20

**Authors:** Liu Chengcheng, Qi Wenwen, Gong Ningyue, Zhu Fangyuan, Xu Runtong, Teng Zhenxiao, Xu Fenglei, Qin Yiming, Zhao Miaoqing, Li Xiaoming, Xia Ming

**Affiliations:** ^1^Department of Central Laboratory, Shandong Provincial Hospital Affiliated to Shandong First Medical University, Jinan, China; ^2^Department of Otolaryngology, Shandong Provincial Hospital Affiliated to Shandong First Medical University, Jinan, China; ^3^Cheeloo College of Medicine, Shandong Provincial Hospital, Shandong University, Jinan, China; ^4^College of Chemistry, Chemical Engineering and Materials Science, Shandong Normal University, Jinan, China; ^5^Department of Pathology, Shandong Provincial Hospital Affiliated to Shandong First Medical University, Jinan, China

**Keywords:** head and neck squamous cell carcinoma, recurrence, immune score, stromal score, immune infiltrating

## Abstract

Head and neck squamous cell carcinomas (HNSCC) are still one of the most common malignant tumors in China, with a high metastasis rate and poor prognosis. The tumor immune microenvironment can affect the occurrence, development and prognosis of tumors, but the underlying mechanism is still unclear. In this study, we tried to describe the correlation between the recurrence of HNSCC and the tumor microenvironment (TME). The expression data [estimate the level of tumor stromal and immune infiltration, expression data (ESTIMATE)] algorithm was used to identify and estimate highly correlated stromal cells, immune cells, and prognostic scores in 116 samples of head and neck cancer patients from The Cancer Genome Atlas (TCGA) dataset. The functional enrichment analysis and protein-protein interaction (PPI) networks of differential expressed genes (DEGs) were constructed. Subsequently, the abundance of various infiltrating immune cells was estimated with the tumor immune estimation resource (TIMER) and the infiltration pattern of immune cells were explored in HNSCC. A total of 407 immune-related genes were identified to involve in the TME. We found that CCR5, CD3E, CD4, and HLA -DRB1 were the most obvious DEGs and the dendritic cells (DCs) showed the highest abundance in the TME of HNSCC. In addition, the unsupervised cluster analysis determined 10 clusters of immune infiltration patterns, and indicated that immune infiltrated CD4 + T and B cells may be related to the prognosis of HNSCC. In conclusion, our research determined the list of immune genes and immune infiltrating cells related to the prognosis of HNSCC, and provided a perspective for HNSCC evolution, anti-tumor drugs selection, and drug resistance research.

## Introduction

Head and neck squamous cell carcinomas (HNSCC), mainly including oral cavity, oropharyngeal, laryngopharyngeal, laryngeal cancers, and hypopharyngeal, are the sixth most common malignant tumors with a 5-year overall survival rate of less than 50% (Kanazawa et al., 2019; McDermott and Bowles, 2019). These dismal malignant tumors affect more than 6,00,000 patients worldwide annually, and nearly half of the newly diagnosed cases are found at an advanced stage (Brenner et al., 2014). Though significant development in multimodal therapeutic strategies, including surgery, radiotherapy, chemotherapy, immunotherapy, and combined treatment, the 5-year survival rate of HNSCC has improved slightly in the past decade due to the resistant nature of tumor cells to chemoradiotherapy (Paver et al., 2020). Recently, emerging genomic evidence indicates that significant immunosuppression and heterogeneous characteristics of HNSCC play vital roles in disease treatment and recurrence (Bhat et al., 2021). However, the detailed roles and underlying mechanisms of tumor heterogeneous on the relapse of HNSCC have not been fully understood.

Disturbance of the tumor microenvironment (TME) is one of the key causes of local immune dysfunction. It establishes a stable ecological environment and space for cancer cells, a variety of endothelial cells, immune cells, and extracellular components (Hanahan and Coussens, 2012; Quail and Joyce, 2013). More and more evidences show that TME is crucial in the occurrence and even recurrence of HNSCC. It has an adverse effect on the prognosis by promoting the evolution of aggressive HNSCC and resistance to chemo- or radio-treatment (Sharma et al., 2017). An in-depth understanding of the influencing factors of TME and the highly complex tumor-stromal interactions can promote the discovery of new therapeutic interventions for HNSCC. Therefore, identifying the immune genes and active types of immune cells related to TME can help to clarify the general mechanism of HNSCC immunosuppression.

Recent studies show that the regulation of the immune system played an important role in the prognosis of cancer patients, and tumor molecular profiling could help to predict clinical outcomes and determine the targets for treating tumors (Michot et al., 2016; Miyauchi et al., 2019; Bruni et al., 2020). It also revealed the prognostic characteristics of some genes in detail, which played an important role in the survival prediction of HNSCC (Zhou et al., 2019). Recently, researchers have identified some important immune checkpoint components that promote the tumor cells to evade from the immune surveillance, such as cytotoxic T lymphocyte-associated protein 4 (CTLA-4) and programmed cell death protein 1 (PD-1)/programmed cells Death ligand 1 (PD-L1) (Brunner-Weinzierl and Rudd, 2018; Lee et al., 2019). Subsequently, novel strategy for cancer therapy of the checkpoint immunosuppression was applied for multiple malignant tumors, including HNSCC. However, checkpoint blocking immunotherapy is not effective for all patients, and the observed effective rate is only in the range of 16–25% (Medina and Adams, 2016). Therefore, there is an urgent need for an immune-related prognostic feature that can predict not only the survival period, but also the immunotherapy response of different patient groups.

In this study, we constructed an immune-related prognostic feature with patient information from The Cancer Genome Atlas (TCGA) data set, and further verified its prognostic value. In addition, we also determined the relationship between the genetic characteristics, TME, and the prognosis of patients with recurrent HNSCC. We further obtained four new prognostic molecular biomarkers, which was closely related to the immune microenvironment of HNSCC. Finally, we verified the expression of these four genes in tumors in a cohort study. The purpose of this study is to provide a new immune-related molecular biomarker of HNSCC, which can more effectively predict the prognosis of HNSCC patients and is closely related to the immune microenvironment.

## Materials and Methods

### Reagants

The rabbit polyclonal to CCR5 (Cat. No. ab7346), rabbit monoclonal to CD3 epsilon (Cat. No. ab237721), and CD4 (Cat. No. ab183685) were purchased from abcam (Cambridge, United Kingdom). The mouse monoclonal to GAPDH (Cat. No. ab8245) and HLA-DRB1 (Cat. No. ab215835) were also purchased from abcam (Cambridge, United Kingdom).

### Gene Expression Data Extraction

The Cancer Genome Atlas dataset is downloaded from UCSC Xena.^1^ RNA sequencing (RNA-seq) data is downloaded from the TCGA data portal. Then convert the FPKM value of the fragment to the value of TPM, including tumor grade and survival information. Our study included 116 tumor samples (Larynx HNSC) with complete prognostic information. We defined patients with new tumors new tumor events occurring after surgery to be recurrent HNSCC. Supplementary Table 1 summarizes the characteristics of the patients in the training and validation cohort.

### Calculation of Stromal and Immune Score

The expression data [estimate the level of tumor stromal and immune infiltration, expression data (ESTIMATE)] analysis by the “estimate” R software package was used to evaluate the stromal and immune scores in stromal cells and immune cells in recurrent HNSCC tissues (Van Landuyt et al., 2011). According to this algorithm, the tumor purity is calculated.

### Differential Expression Analysis

According to the ESTIMATE analysis, we divided all patients into a high/low immune score group and a high/low stromal score group. Then, by using the “limma” package to analyze the differential expression between different immune groups, the selection criteria are log2∣fold change∣ greater than 1 and adj.p less than 0.05. The corresponding heat maps and cluster maps were generated using the “pheatmap” package.

### Survival Analysis

Kaplan–Meier analysis was performed to screen HNSCC with recurrent characteristics. The survival curves were also applied to illustrate the relationship between the expression levels of these genes and the recurrence of HNSCC.

### Gene Ontology and Kyoto Encyclopedia of Genes and Genomes Analysis

Functional enrichment analysis was performed by the “clusterProfiler” package to determine the functions and pathways of the screened genes (Yu et al., 2012). Functional enhancements to gene ontology (GO) terms include cell component (CC), biological processes (BPs), molecular function categories (MF), and the Kyoto Encyclopedia of Genes and Genomes (KEGG) approach in the Kyoto Protocol. The cut-off value was false discovery rate<0.05.

### Protein-Protein Interaction Network Construction

A total of 407 genes were obtained and mapped from the STRING database. The selected interaction score was greater than 0. The protein-protein interaction (PPI) network between the obtained genes was also analyzed. Search tool STRING^2^ that retrieves interacting genes was used to predict the PPI network of immune-related genes (Ke et al., 2019). An interactive composite score greater than 0.4 is considered statistically significant. Use Cytoscape’s Molecular Complex Detection (MCODE) plug-in to perform topological clustering of the network, where the recognition MCODE score is greater than 5, the degree cutoff is equal to 2, the node score cutoff is equal to 0.2, the maximum depth is equal to 100, and the *k* score is equal to 2 (Liu et al., 2019).

### Immune Cell Infiltration Analysis

The abundance of the six infiltrating immune cells in recurrent HNSCC was calculated by the tumor immune estimation resource (TIMER) algorithm (a calculation method). The infiltrating cells mainly included dendritic cells (DCs), neutrophils, B cells, macrophages, CD4 + and CD8 + T cells. Then, the patients were divided into *k* clusters by unsupervised cluster analysis of these infiltrated immune cells based on *k*-means (Seo et al., 2018; Yang et al., 2019). In this analysis, we divide the patients into 10 Clusters, and investigated the association of different clusters with recurrence of HNSCC.

### Quantitative Real Time-PCR

In this study, 30 HNSCC patients who underwent tumor resection but did not receive adjuvant therapy such as radio- or chemotherapy before surgery at Shandong Provincial Hospital Affiliated to Shandong First Medical University (Shandong, China) participated in this project in accordance with the Declaration of Helsinki. The tumor and control tissues are collected in RNase-free eppendorf tubes, and stored in −80°C. Then total RNA was isolated according to the manufacturer’s protocol (RNeasy Mini Kit, Qiagen). The extracted RNA was then reverse transcribed using Superscript R reverse transcriptase. Quantitative real time-PCR (qRT-PCR) was performed using cDNA as a template with Platinum SYBR Green (Invitrogen). To obtain the best sensitivity and specificity of the primers, the cycle length of different PCR groups was adjusted to 40 cycles, and the annealing temperature was adjusted between 56 and 64°C. To analyze the qRT-PCR results, the 2-ΔΔCt method was used, and β-actin served as a housekeeping gene. All PCRs were performed in triplicate. Specific primer sequences are recorded below: CCR5-forward primer (AGGAATCATCTTTACCAGAT), reverse primer (ATGACAAGCAGCGGCA); CD3E-forward primer (TCCCAACCCAGACTATGAGC), reverse primer (CAAGACTAGCCCAGGAAACAG); CD4-forward primer (AGTCCAAGGGGTAAAAACAT), reverse primer (AAGGAG AACTCCACCTGTTC); HLA-DRB1-forward primer (AGAG CTTCACAGTGCAGCGG), reverse primer (GTCTTCTCTT CCTGGCCATT); and GAPDH-forward primer (GGGAAA CTGTGGCGTGAT), reverse primer (GAGTGGGTGTCG CTGTTGA).

### Immunohistochemistry

All nasal biopsy specimens embedded in paraffin were cut into 4 um sections using a Leica microtome (Leica, Wetzlar, Germany). After dewaxing with xylene, a modified citrate buffer was used for thermally induced epitope repair. The samples were blocked with 10% goat serum and then incubated with the primary antibody overnight at 4°C. Modified horseradish peroxidase (HRP) technology was used to observe specific protein expression. High-resolution images were obtained on an Olympus digital electron microscope (Olympus, Tokyo, Japan).

### Western Blot

The tumor and control tissues are lysed with cell lysis buffer with 1 mM phenylmethanesulfonyl fluoride (PMSF) and 1×protease inhibitor cocktail (Roche). For Western blot analysis, the protein sample concentration was adjusted to 40 μg/μl, separated by SDS-PAGE, then switched to a PVDF membrane, and incubated with the corresponding primary antibody at 4°C overnight. The next day, the corresponding secondary antibody was incubated for 1 h at room temperature. An ECL system (Cell Signaling Technology) was used to detect protein signals. The GAPDH protein in the whole protein was used as an internal control.

### Statistical Analysis

The ‘‘ggplot2’’ R package was used for data visualization, and all statistical analysis was performed in R (version 3.6.1).^3^ The Kaplan–Meier method was used to generate visual survival curves, and all survival curves were generated by the “survminer” R package. We performed the Benjamini–Hochberg method to convert the *P*-value of a gene into FDR for the analysis of differential expressed genes (DEGs). The “pheatmap” R package was applied for heat map generation and visualization. For multiple groups, one-way analysis of variance (ANOVA) was selected as the parameter method for comparing means, and the Kruskal–Wallis test analysis was performed by the non-parametric method. The Wilcoxon test was used to compare non-normally distributed variables. The unpaired Student *t*-test was used to compare normally distributed variables. The Shapiro–Wilk normality test was used to test the normality of the variables. All tests are two-tailed tests, and a *P*-value of less than 0.05 was considered statistically significance.

## Results

### Association of Immune and Stromal Scores With Pathological Characteristics of Recurrent HNSCC

**FIGURE 1:**
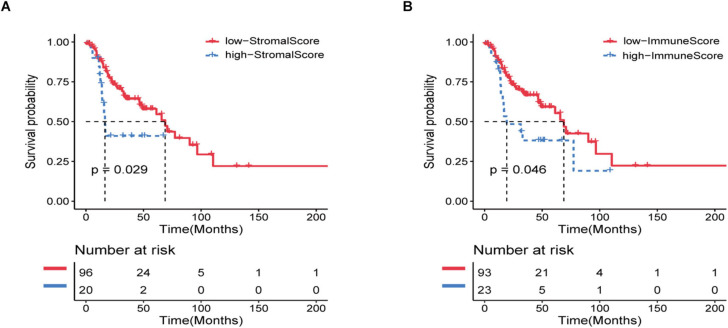
Immune and stromal scores are significantly related to the prognosis of patients with recurrent HNSCC. Based on the immune/stromal scores, recurrent HNSCC patients were divided into high/low score groups. **(A)** Kaplan–Meier curve of the high and low immune score groups. **(B)** Kaplan–Meier curve of high and low stromal score groups.

A total of 116 eligible HNSCC data, including clinicopathological and prognostic informations, were extracted from the TCGA database. In general, 65.5% of the patients were over 60 years old, 43.1% were alive, and 75.9% were in stage III/IV at the time of diagnosis (Supplementary Table 1). Using ESTIMATE analysis, immune score and stroma score were performed according to age, tumor grade, and survival status and divided all patients into high- and low-score groups (Supplementary Table 1). Kaplan–Meier analysis was performed and the result indicated that the immune and stroma scores were obviously correlated with the prognosis of recurrent HNSCC patients (Figures 1A,B). In addition, we also tried to explore the relationship between immune and stromal scores and the age, tumor grade, and treatment methods of patients with recurrent HNSCC. The analysis showed that immunity and stromal scores have a certain relationship with age and tumor grade, but there is no significant correlation with treatment (Supplementary Figure 1).

### Identification of Immune-Related Genes in Recurrent HNSCC

**FIGURE 2:**
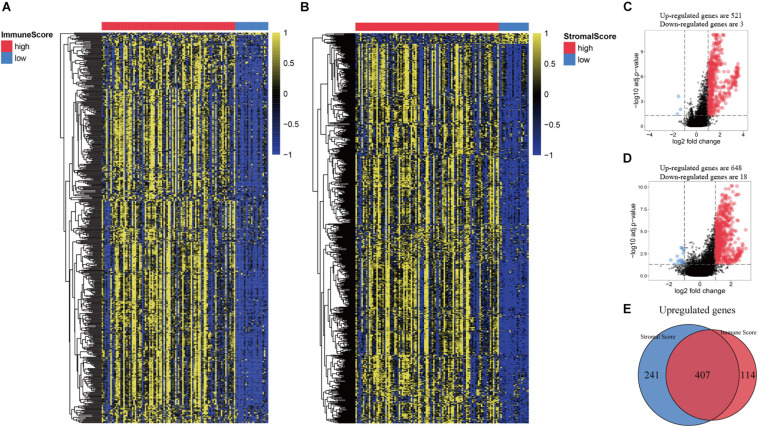
Comparison of gene expression profiles based on immune score and stromal score in recurrent HNSCC. **(A)** Heat map of genes that are significantly expressed differently based on immune scores. **(B)** Heat map of genes that are significantly differently expressed based on stromal scores. **(C)** Volcano map of differential expressed genes (DEGs) according to the immune score. **(D)** Volcano map of DEGs according to the stromal scores. **(E)** Venn diagram analysis of abnormally expressed genes according to the immune and stromal scores.

All patients were divided into a high or low immune score group and a high or low stroma score group according to the results of ESTIMATE analysis. Subsequently, gene differential expression analysis was performed to identify the DEGs between the high or low groups of immune score and stroma score. The heat map shows the gene expression pattern of DEGs between the high and low immune score or stroma score groups (Figures 2A,B). Then, gene expression levels in the high and low immune score groups were compared. There was 521 up-regulated genes and 3 down-regulated genes in the high immune score group (Figure 2C). Similarly, 648 genes were up-regulated and 18 genes were down-regulated in the high stroma score group (Figure 2D). As shown in the Venn diagram (Figure 2E), we selected the up-regulated overlapping genes as candidate immune-related genes (407 up-regulated genes), because there is no overlap of the down-regulated genes in the immune group and the stroma group. These crossover genes will be used for subsequent analysis.

### Module-Related Central Gene Network

**FIGURE 3:**
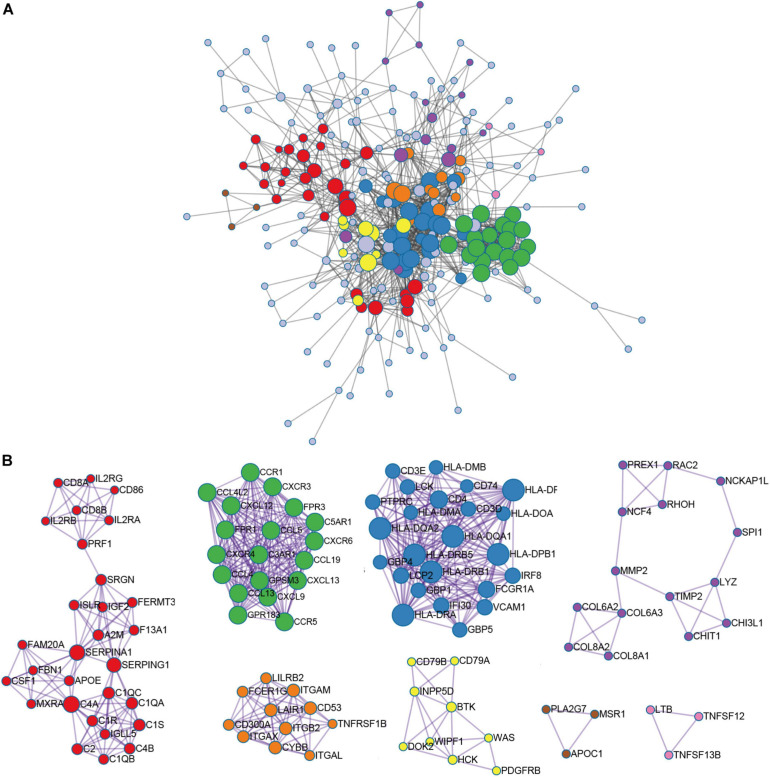
Module-related central genes network in recurrent HNSCC. **(A)** STRING database and Cytoscape software were used to construct the protein-protein interaction (PPI) network module. In the PPI network, the color of the node reflects the logarithmic (FC) value, the size of the node indicates the number of interacting protein. **(B)** Through Molecular Complex Detection (MCODE) analysis, eight modules with at least three nodes are determined. The more a gene is associated with other genes in the network, the more relevant the module is.

In order to further identify the DEGs related to TME in HNSCC, we constructed a modular-centric gene network. We also calculated a PPI network with 212 nodes and 2,016 edges to explore the interaction between the candidate immune-related genes of tumor prognosis (Figure 3A). It can be seen that the more a gene is associated with other genes in the network, the more relevant the module is. In addition, MCODE analysis identified 8 modules with at least 3 nodes (Figure 3B). Among the 8 modules, the red module had 29 nodes and 372 edges, which scored the highest among these modules, and the smallest brown and pink module had only 3 nodes and 3 edges. To some extent, there are fewer nodes with a large number and more nodes with a small number, which is consistent with the characteristics of biological networks.

### Function Richness Analysis of Immune-Related Genes in Recurrent HNSCC

**FIGURE 4:**
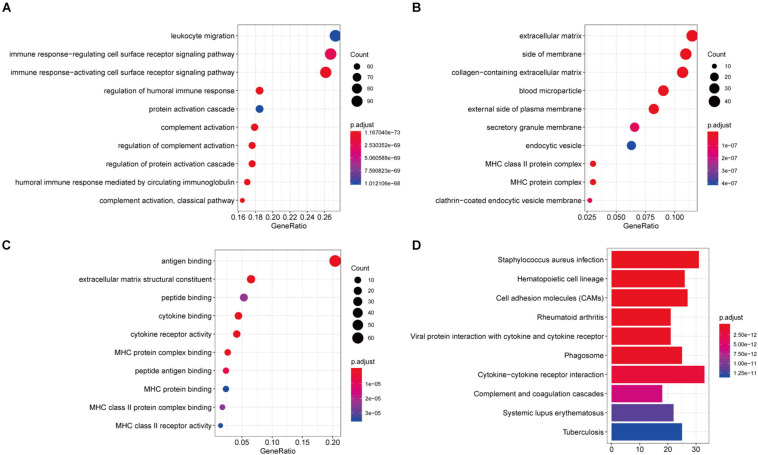
Function richness analysis of immune-related genes in recurrent HNSCC. **(A)** The biological process (BP) of genetic ontology (GO) of prognostic immune-related genes. **(B)** Cellular components of the gene ontology (GO) of prognostic immune-related genes. **(C)** The molecular function (MF) of the GO of prognostic immune-related genes. **(D)** Kyoto Encyclopedia of Genes and Genomes (KEGG) pathway enrichment analysis of prognostic immune-related genes. The color of the bar indicates –log10 (*p*-value). The intersection of the KEGG pathway and GO Term is enriched by the number of genes.

In order to explore the potential molecular mechanisms of 407 immune-related genes, we performed a functional enrichment analysis of these genes. As shown in Figure 3 and Supplementary Table 2, the BP of GO analysis shows that immune-related genes are mainly enriched in the BP of leukocyte migration, immune response-regulating cell surface receptor signaling pathway and immune response-activating cell surface receptor signaling pathway (Figure 4A). In the CC group genes are mainly enriched in the extracellular matrix, side of membrane, and collagen-containing extracellular matrix (Figure 4B). MF enriches antigen binding, extracellular matrix structural constituent, peptide binding (Figure 4C). In addition, KEGG analysis showed that immune-related genes are related to staphylococcus aureus infection, hematopoietic cell lineage, cell adhesion molecules (CAMs), rheumatoid arthritis, viral protein interaction with cytokine and cytokine receptor, phagosome, and cytokine-cytokine receptor interaction (Figure 4D and Supplementary Table 2). The above results indicate that immune-related genes are related to the extracellular stroma, bacterial infections, and cell interactions of the TME.

### Survival Analysis of Immune-Related Genes in Recurrent HNSCC

**FIGURE 5:**
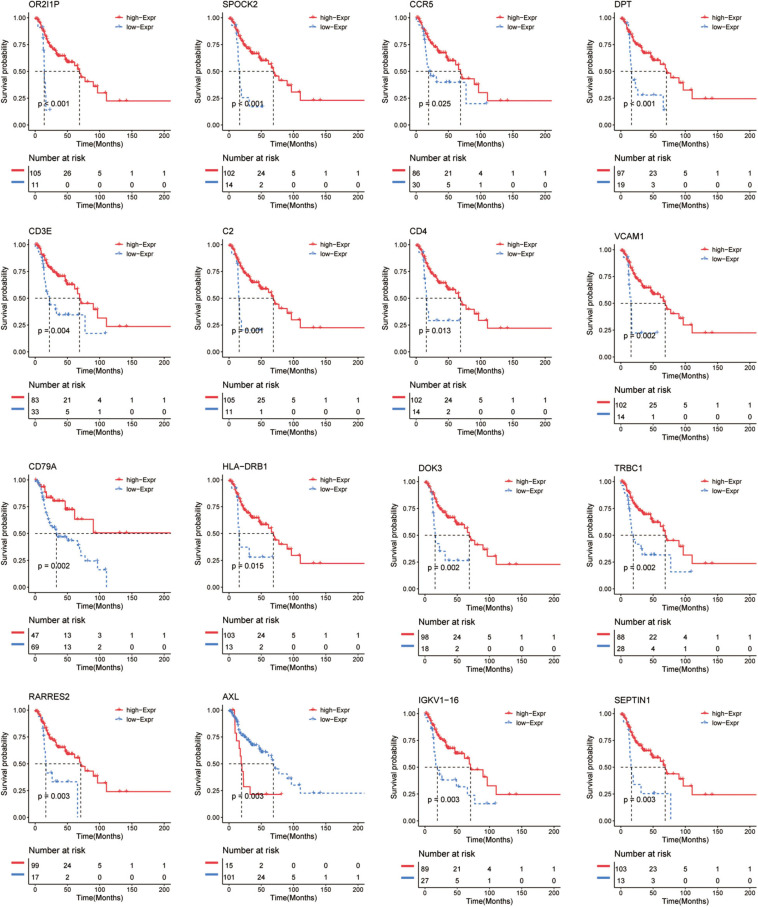
Kaplan–Meier survival curve shows the survival analysis of immune-related genes in recurrent HNSCC. The expression level of 16 immune-related genes to affect the overall survival are selected randomly, and compare the overall survival of the high and low gene expression groups. The red and blue dashed lines represent the upper and lower limits of the 95% confidence interval for gene expression, respectively.

To further investigate the prognostic value of these immune-related genes, Kaplan–Meier analysis was performed and survival curves were illustrated to further investigate the relationship between the expression levels of these immune-related genes and the survival of recurrent HNSCC patients. The results indicated that a total of 206 genes were significantly associated with the survival of the recurrent HNSCC among which, OR2I1P, SPOCK2, CCR5, DPT, CD3E, C2, CD4, VCAM1, CD79A, HLA-DRB1, DOK3, TRBC1, RARRES2, AXL, and IGKV1-16 are the most significant prognostic factors in recurrent HNSCC patients (Figure 5 and Supplementary Table 3). We therefore selected these prognostic immune-related genes for further analysis.

### The Immune Landscape of Recurrent HNSCC TME

**FIGURE 6:**
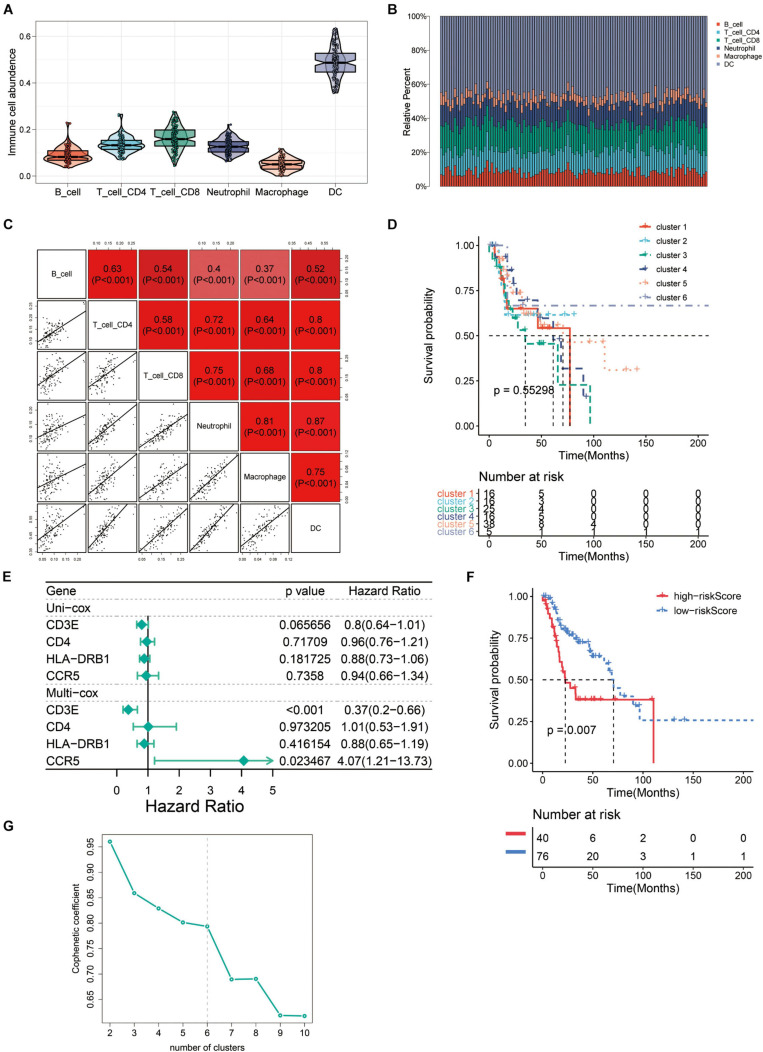
The immune landscape of recurrent HNSCC tumor microenvironment (TME). **(A)** Abundance of immune infiltrating cells in recurrent HNSCC. **(B)** Stacked bar graph of samples arranged in the order of cluster assignment, and unsupervised clustering of all samples based on the proportion of immune cells. **(C)** Correlation stroma of the abundance of six immune cells. **(D)** Summary of survival analysis of patients in different groups. **(E)** Uni-Cox and Multi-Cox regression analysis of CCR5, CD3E, CD4, and HLA-DRB1 four genes. **(F)** Survival analysis of CCR5, CD3E, CD4, and HLA-DRB1 four genes in the high-risk group and the low-risk group. **(G)** Optimal number of clusters by unsupervised clustering analysis.

In order to evaluate the TME in recurrent HNSCC, we estimated the abundance of tumor infiltrating immune cells, and explored the impact of these infiltrating immune cells on the clinical outcome in recurrent HNSCC patients. Among the six types of immune cells, DCs have the highest abundance in the microenvironment of HNSCC (Figure 6A). Figure 6B summarizes the proportion of immune cells in each cluster. Subsequently, we analyzed the abundance matrix of different tumor infiltrating immune cells and found that the abundance of different infiltrating cells was weakly correlated (Figure 6C and Supplementary Table 4). Then, we further studied the relationship between different immune cell infiltration patterns and recurrence HNSCC survival. As shown in Figure 6D, cluster 3, defined by high levels of CD4 + T cells and low levels of B cells is associated with a better prognosis, while cluster 5 (defined by low levels of CD4 + T cells and low levels of B cells) is associated with better prognosis. Cell definition had the worst outcomes. The above results indicate that changes in the abundance of tumor infiltrating immune cells may be related to the prognosis of recurrent HNSCC.

### Expression Verification of Four Screened Immune-Related Gene

In order to find the genes that are most critical to the TME in HNSCC, we combined the genes located in the middle node of the PPI network (Figure 3A) and the *p*-value less than 0.05 in the survival analysis to screen out four genes CCR5, CD3E, CD4, and HLA-DRB1. After grouping these four genes into high/low risk scores, the survival risk assessment was performed again, and it was found that CCR5, CD3E, CD4, and HLA-DRB1 genes significantly affected the survival risk of patients (Figures 6E,F and Supplementary Table 5). Considering the unique distribution of individual infiltrating immune cells, all patients were subjected to unsupervised clustering of *k*-means algorithm according to the proportion of immune cells, and the optimal number of clusters was ten (*k*-means = 10, Figure 6G and Supplementary Table 6).

**FIGURE 7:**
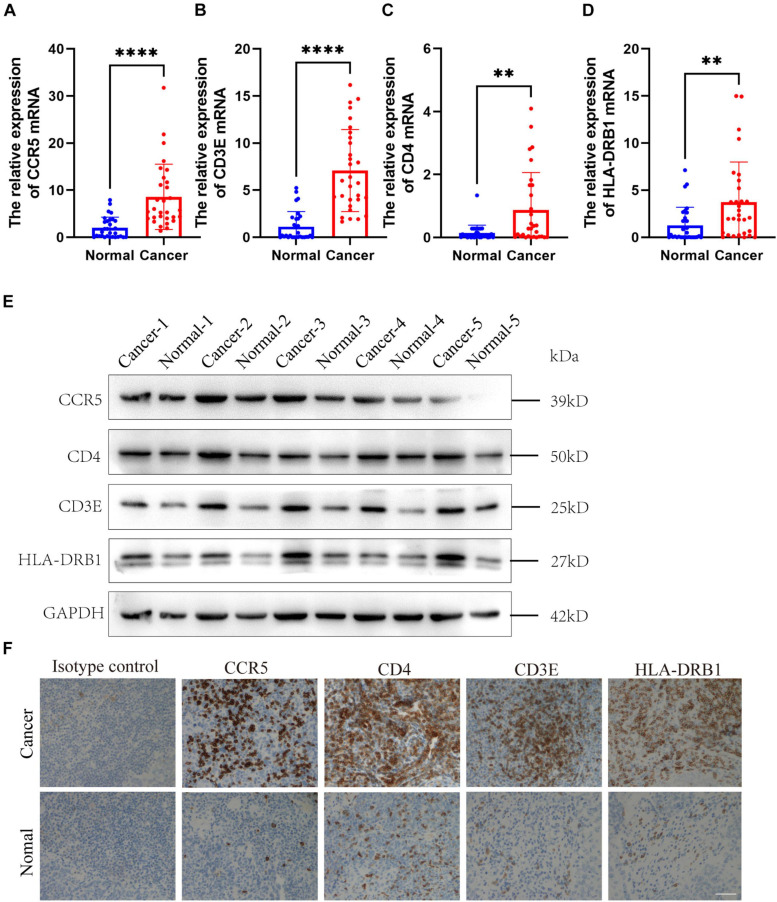
Expression verification of four screened immune-related gene. **(A–D)** The mRNA levels of CCR5, CD3E, CD4, and HLA-DRB1 in HNSCC samples. **(E)** The protein levels of CCR5, CD3E, CD4, and HLA-DRB1 in HNSCC samples. **(F)** Representative immunohistochemicalimages of CCR5, CD3E, CD4, and HLA-DRB1. Original magnification: ×200. Bar = 50 um. **P < 0.01, ****P < 0.0001.

To further verify the results, the relative mRNA expressions of CCR5, CD3E, CD4, and HLA-DRB1 in HNSCC tumor tissue and adjacent non-tumor tissues were tested. The results showed that the expression of CCR5, CD3E, CD4, and HLA-DRB1 mRNAs in tumor tissues was significantly higher than that of normal tissues adjacent to the tumor (Figures 7A–D). Our western blot analysis showed that compared with normal tissues, HNSCC tissues showed relatively higher expression levels of CCR5, CD3E, CD4, and HLA-DRB1 proteins (Figure 7E). In addition, IHC analysis was performed to determine the protein expression levels of CCR5, CD3E, CD4, and HLA-DRB1 in HNSCC. From the immunostaining, we can observe that the expression of these four proteins is increased in tumor tissues compared with neighboring normal tissues (Figure 7F). The above results all indicate that CCR5, CD3E, CD4, and HLA-DRB1 are highly expressed in HNSCC tumor tissues.

## Discussion

Head and neck squamous cell carcinomas is one of the most common malignant tumors in the world, with a mortality rate of 40–50% (McDermott and Bowles, 2019). Considering the difficulty of early diagnosis, most HNSCC patients are already at an advanced stage at diagnosis. In these advanced HNSCC patients, local metastasis and recurrence of cancer cells are common and in need of improved treatment (Paver et al., 2020). The prognosis is even worse for relapsed HNSCC patients with progressive disease after platinum therapy (Burtness et al., 2019). In order to solve these difficulties faced by HNSCC treatment, new treatment methods have emerged. Cetuximab is a monoclonal antibody (mAb) that targets epidermal growth factor receptor (EGFR), which was introduced into platinum-based therapy for HNSCC (Cohen et al., 2019). Immunotherapy attracts people’s attention due to it fights tumors by activating one’s own immune cells (Poggio et al., 2019). Although immunotherapy provides HNSCC patients with a cancer treatment option, and HNSCC have a wide range of immune-related genomic features, the immune molecular mechanism of HNSCC is still little known. Therefore, we use TCGA databases to screen prognostic immune-related biomarkers through ESTIMATE and TIMER algorithms, and provide new insights for the treatment of HNSCC.

Increasing studies have shown that the regulation of TME is not only related to immunotherapy but also related to the prognosis of tumor patients (Chen et al., 2019; Liu G. et al., 2021). To ESTIMATE was used to model and test the estimation of stromal cells and immune cells in HNSCC tissue to calculate the purity of the tumor, stromal, and immune score through expression profiles, and give an overall view of the TME. We found that the immunity and stromal scores were significantly related to the prognosis of HNSCC patient. Then, we performed differential expression analysis between the high/low immune score groups and between the high/low stroma score groups, and identified 407 immune-related genes (Figure 2). In our results, almost all DEGs are up-regulated, we speculate that the possible reason is that tumor cells have faster energy metabolism and proliferation, which leads to more active gene transcription. Subsequently, GO and KEGG were used to analyze these immune-related genes to reveal their functions. As expected, most of the immune-related genes are involved in immune processes (Figure 3). These results indicate that the extracellular matrix in the TME has a strong correlation with immune infiltrating cells. In addition, we constructed a PPI module, which is used to reveal the relationship and gene function between DEGs (Figure 4). The nodes with rich connectivity in the module are also related to immune/inflammatory response.

To better explore the impact of immune infiltrating cells and extracellular matrix related genes on prognosis, we performed Kaplan–Meier analysis to explore potential regulatory mechanisms. Survival analysis was performed according to the 407 differential immune-related genes and 206 genes were closely correlated to the survival rate of the recurrent HNSCC patients (Figure 5). To further explore the relationship between these DEGs and immune cells, we performed the TIMER method to estimate the proportion of different immune infiltrating cells. Furthermore, unsupervised cluster analysis is also used to identify the immune infiltrating patterns in HNSCC. The results were consistent with other studies that monocyte immune cells such as B cells, CD4 + T cells, CD8 + T cells, macrophages, and natural killer (NK) cells played a key role in tumor immunity and were also related to tumor prognosis in HNSCC patients (Djaldetti and Bessler, 2014; Tamminga et al., 2020).

In order to investigate effects of the immune-related genes on HNSCC prognosis, we further screened these prognostic immune-related genes and explored the potential regulatory mechanisms. A total of four genes (CCR5, CD3E, CD4, and HLA-DRB1) were identified that related to the immune matrix and immune cells. We found that HNSCC patients with high expression of these four immune-related genes (*P* > 0.05) had a worse prognosis (*P* < 0.05). To further verify the accuracy, we conducted verification in HNSCC patient tissues, and found that the mRNA expression of CCR5, CD3E, CD4, and HLA-DRB1 were significantly upregulated in HNSCC tissues comparing with that of the controlled tissues. The protein levels of these genes were also identical to that (Figure 7). The immune microenvironment can reflect the immune status of tumor patients to a certain extent, which provides a reference for us to treat tumors more accurately. Many studies have shown that CCR5, CD3E, CD4, and HLA-DRB1 play an important role in the development of tumors. Anti-CCR5 therapy in cancer patients can effectively target tumor immune cells in colorectal cancer metastasis (Jiao et al., 2019). As an indicator of bladder cancer TME regulation, CD3E is closely related to immune infiltration (Liu Y. et al., 2021). The tumor antigen-specific CD4 + T cells in cancer immunity are a key strategy for tumor prognosis and treatment (Protti et al., 2014). HLA-DRB1 expression can predict the prognosis of patients with non-small cell lung cancer who receive PD-1/PD-L1 immune checkpoint blockade (Correale et al., 2020). In our study, a total of 10 clusters were formed, and further survival analysis showed that changes in the abundance of CD4 + T cells and B cells may be the main prognostic factors for HNSCC. In summary, these results indicate that the heterogeneity of immune infiltration in HNSCC can be used as a prognostic indicator and therapeutic target for immunotherapy.

**FIGURE 8:**
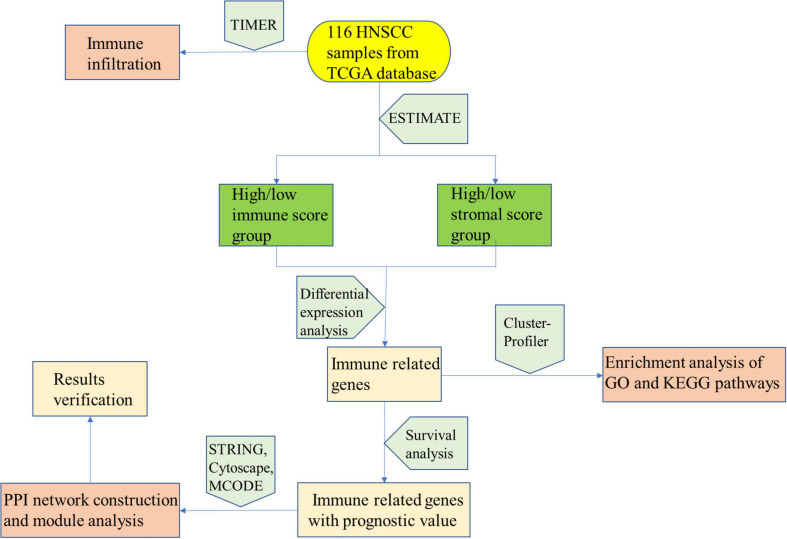
Analysis process structure diagram.

In conclusion, as shown in Figure 8, our analysis process was mainly performed based on the ESTIMATE algorithm to calculate the immune as well as stromal scores and further detect the correlations between the immune/stromal scores and clinical parameters. According to the immune/stromal scores, we divided the HNSCC patient obtained in the TCGA database into high or low score immune group and matrix group to determine the DEGs. Subsequently, the results of functional enrichment analysis showed that these genes are mainly involved in the immune/inflammatory response, and the PPI network further demonstrated this discovery. Finally, we calculated the abundance of each type of immune cells in the TME to explore the relationship between immune scores and immune cells. The above analysis enables us to better understand the TEM and immune characteristics of HNSCC, and helps us to predict the prognosis of HNSCC patients through immune-related genes.

## Data Availability Statement

The datasets presented in this study can be found in online repositories. The names of the repository/repositories and accession number(s) can be found in the article/Supplementary Material.

## Ethics Statement

The studies involving human participants were reviewed and approved by the Biomedical Research Ethic Committee of Shandong Provincial Hospital. The patients/participants provided their written informed consent to participate in this study.

## Author Contributions

LC drafted the manuscript, performed the experiments, and analyzed the data. QW, GN, ZF, XR, TZ, XF, and QY designed the study and analyzed the data. XM, LX, and ZM revised the manuscript. All authors contributed to the article and approved the submitted version.

## Conflict of Interest

The authors declare that the research was conducted in the absence of any commercial or financial relationships that could be construed as a potential conflict of interest.

## Publisher’s Note

All claims expressed in this article are solely those of the authors and do not necessarily represent those of their affiliated organizations, or those of the publisher, the editors and the reviewers. Any product that may be evaluated in this article, or claim that may be made by its manufacturer, is not guaranteed or endorsed by the publisher.

## Footnotes

1
https://xenabrowser.net/

2
https://string-db.org/

3
https://www.r-project.org/

## Supplementary Material

Supplementary Figure 1 Correlation between immune and stromal scores and characteristics of patients with recurrent head and neck squamous cell carcinomas (HNSCC). **(A)** Immune scores’ distribution for ages <60 and ≥60. **(B)** Immune scores’ distribution of tumor grade. **(C)** Stromal scores’ distribution for ages<60 and ≥ 60. **(D)** Stromal scores’ distribution of tumor grade. **(E)** The associations of immune and stromal scores with the treatment of recurrent HNSCC patients.

Supplementary Table 1 The characteristics of the patients in the training and validation cohort.

Supplementary Table 2 Gene ontology (GO) and Kyoto Encyclopedia of Genes and Genomes (KEGG) analysis of 407 immune-related genes.

Supplementary Table 3 Survival analysis of immune-related genes in recurrent head and neck squamous cell carcinomas (HNSCC).

Supplementary Table 4 The abundance stroma of different tumor infiltrating immune cells in recurrent HNSCC.

Supplementary Table 5 Survival analysis of CCR5, CD3E, CD4, and HLA-DRB1 genes in the high-risk group and the low-risk group.

Supplementary Table 6 Optimal number of clusters by unsupervised clustering analysis.
