# Supplementary figures and images for: Identification of the Immune-Related Genes in Tumor Microenvironment That Associated With the Recurrence of Head and Neck Squamous Cell Carcinoma
Source: Front Cell Dev Biol. 2021 Aug 20;9:723721. doi: 10.3389/fcell.2021.723721 (PMC8417745; doi:10.3389/fcell.2021.723721)

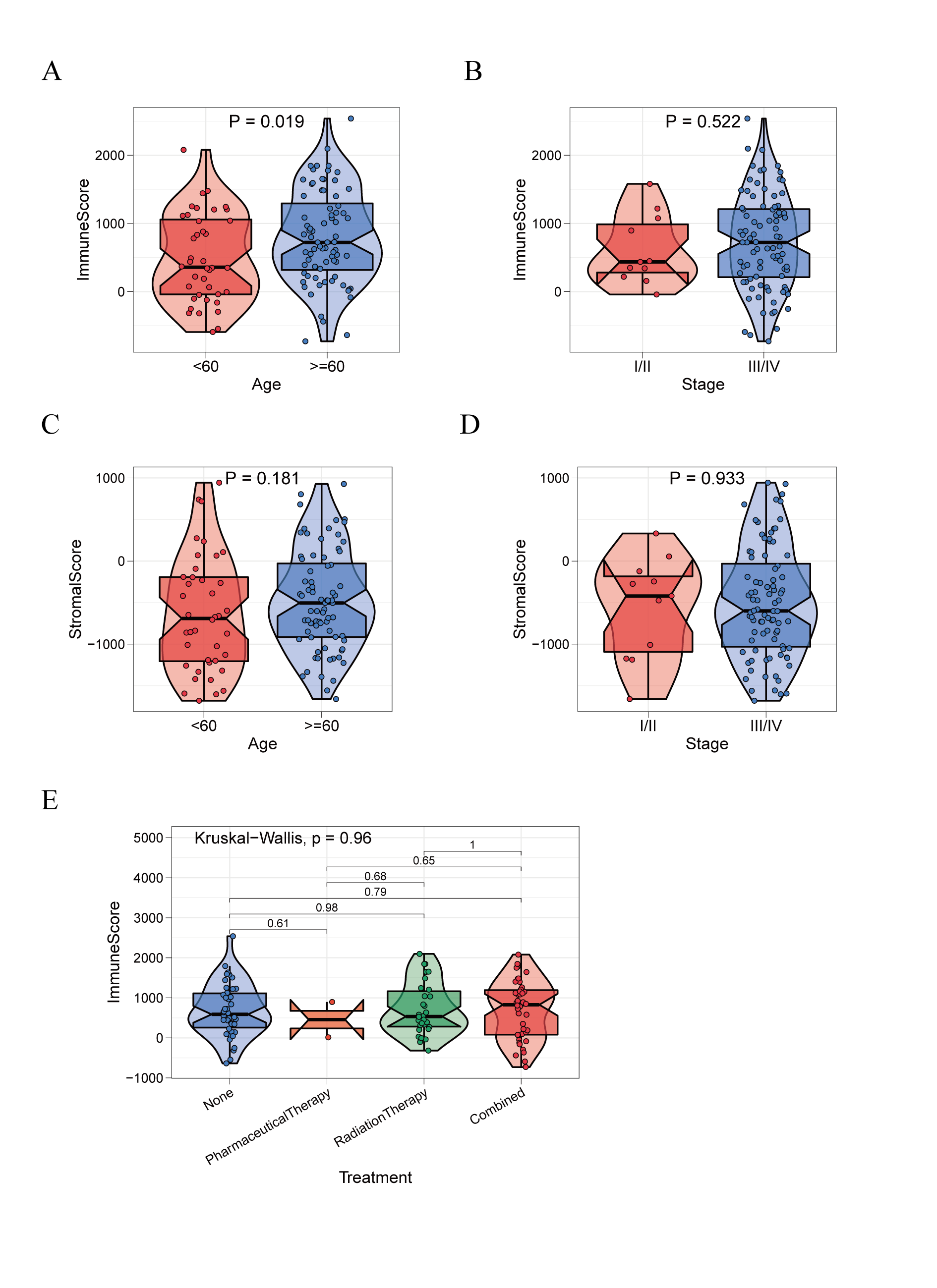

Supplement: Supplementary Figure 1 — Correlation between immune and stromal scores and characteristics of patients with recurrent head and neck squamous cell carcinomas (HNSCC). (A) Immune scores’ distribution for ages <60 and ≥60. (B) Immune scores’ distribution of tumor grade. (C) Stromal scores’ distribution for ages<60 and ≥ 60. (D) Stromal scores’ distribution of tumor grade. (E) The associations of immune and stromal scores with the treatment of recurrent HNSCC patients. [file Image_1.TIF]
